# Patient perspectives of prosthetic heart valve choice and anticoagulation in patients with rheumatic heart disease: a semi-quantitative study

**DOI:** 10.3389/fcvm.2026.1810992

**Published:** 2026-06-08

**Authors:** Shownok Saha, Danielle Gelbart, Francesco Pirone, David J. McCormack, Nishith N. Patel

**Affiliations:** 1 Department of Surgery, School of Medicine, Faculty of Medical and Health Sciences, University of Auckland, Auckland, New Zealand; 2 Department of Cardiology, Te Whatu Ora Waikato, Waikato Hospital, Hamilton, New Zealand; 3 Cardiothoracic Surgery, Te Whatu Ora Waikato, Waikato Hospital, Hamilton, New Zealand; 4 School of Health Sciences, University of Waikato, Hamilton, New Zealand

**Keywords:** anticoagulation, prosthetic heart valve, rheumatic heart disease, shared decision making, surgery

## Abstract

**Objective:**

To understand the factors that influence rheumatic heart disease (RHD) patients' prosthetic heart valve choice, their knowledge of anticoagulation and subsequent experience. This is the first RHD-specific data on valve choice, including high-deprivation populations.

**Methods:**

We performed a semi-quantitative, retrospective, cross-sectional survey study using a semi-structured telephone interview of patients (18–60 years) with RHD who had undergone valve replacement surgery from 2015 to 2020 at a single centre. Patients shared their experiences with prosthetic heart valves and warfarin.

**Results:**

138 patients completed or partially completed the survey from a total sample of 218 patients; mean age was 49 ± 10. 133 (61%) of patients had bioprosthetic valves implanted, and 85 (39%) had mechanical valves implanted. Younger and older patients were more likely to have bioprosthetic valves implanted, whereas middle-aged patients were more likely to have mechanical valves implanted. 38 (44%) of bioprosthetic valve patients specified warfarin avoidance, and 31 (62%) of mechanical valve patients specified avoidance of reoperation as their primary reason for choosing their respective valves (from 136 patients). 37 (43%) of bioprosthetic valve patients were informed that the durability of their valves would be at least 10-15 years (from 136 patients). The most helpful information providers regarding valve type were the surgeon and the cardiologist, whereas the GPs and cardiologists were for warfarin management. 64 (47%) had not seen a dentist, and 46 (34%) had not followed up with a cardiologist post-operatively (from 137 patients).

**Conclusion:**

Many young RHD patients receive bioprosthetic valves; surgeons and cardiologists greatly influenced this choice. A uniform evidence-based education programme for health professionals may enhance decision-making around valve choice.

**Key findings:**

A large proportion of young RHD patients receive bioprosthetic valves, and surgeons and cardiologists greatly influenced this choice. Many patients also do not see a dentist or cardiologist following surgery. A uniform evidence-based education programme for health professionals may enhance decision-making around valve choice, and free dental care and robust follow-up pathways would ensure this vulnerable group of patients is not lost to follow-up.

## Background

Rheumatic heart disease (RHD) remains an important cause of death and cardiovascular morbidity in New Zealand, where it affects nearly 1% of the population (1). There is no effective medical therapy for RHD, and surgery remains the mainstay of management. The primary surgical option in adults is surgical valve repair, where possible, and complete replacements with a mechanical or bioprosthetic valve when necessary, as the populations that are most affected by RHD are typically from lower socioeconomic status with reduced access to care; their outcomes with prosthetic valves, especially mechanical, are poorer. However, RHD pathology makes repair difficult (2–5).

Local evidence from New Zealand emphasises that the primary goal of surgical management in rheumatic heart disease (RHD) should be durable valve repair rather than prosthetic replacement, highlighting that patient outcomes on long-term anticoagulation are often suboptimal (2). This is further supported by a New Zealand cohort study of young patients with RHD undergoing mitral valve replacement, which demonstrated that individuals with mechanical prostheses remained within the therapeutic international normalised ratio (INR) range for only 23% of the time over a medium-term follow-up of approximately six years (3). Similarly, a paediatric study involving patients aged less than 25 years with RHD reported that, within five years following mechanical valve implantation, 72% experienced at least one anticoagulation-related hospitalisation (4). These findings are consistent with international data; for example, a large African study analysing over 20,000 INR measurements from 552 patients with mechanical valves found that only 27% of values were within the therapeutic range, with the majority remaining subtherapeutic. The authors emphasised the importance of identifying patients at high risk of poor anticoagulation adherence when considering valve replacement strategies (5).

Despite the poor outcome data of anticoagulation control, considerably less is known about the patient experience of having a prosthetic valve, particularly the factors that influence prosthetic valve choice. The choice of valve prosthesis is a complex shared decision-making process based on the consideration of several factors, including expected durability, potential need for long-term warfarin management, and patient values and preferences. To date, there is no data from RHD patients on experiences with valve choice and anticoagulation. Current international valve guidelines are based on North American and European populations with degenerative valve disease (6); these guidelines may not reflect the circumstances, priorities and values of young RHD patients in New Zealand.

The aim of this study, therefore, was to understand the factors that influence patients' prosthetic heart valve choice, their knowledge of anticoagulation and subsequent experience with prosthetic heart valves and anticoagulation from an RHD population.

## Methods

### Ethics statement

The study was approved by the clinical research committee of Health New Zealand Waikato to meet ethical and legal requirements, and individual consent was waived.

### Study design

We undertook a retrospective semi-quantitative survey of consecutive RHD patients who had undergone valve replacement surgery between 1st January 2015 and 31st December 2020 at Waikato Hospital, Hamilton, New Zealand. The following exclusion criteria were applied: any emergency surgery in which the patient was unable to choose the valve, any patient undergoing infective endocarditis repair.

### Data collection

Patients were identified from the hospital Dendrite Cardiac Surgical Registry. Demographic, angiographic and procedural data were collected using a specific form routinely entered into a validated national database (Patient Analysis & Tracking System; Dendrite Clinical Systems, London, UK). The form includes five sections filled in consecutively by anaesthetists, surgeons, intensive care and high-dependency unit staff, and ward nurses. Data entry is periodically checked for accuracy by independent database managers.

A telephone survey was used with a pre-designed questionnaire (see appendix). Patients were asked to share their experiences with prosthetic heart valves and warfarin in relation to four specific content areas or themes: decision-making, knowledge and education, impact on daily life and patient satisfaction. The survey was performed at a single tertiary centre (Waikato Hospital) for patients who had undergone valve replacement surgery between 1st January 2015 and 31st December 2020.

Patients were called between three and five times if the phone call was not picked up across two weeks. Patients were then asked to consent to the questionnaire over the phone before being walked through the questions. Participation in the study ended on either a partial or full completion of the questionnaire; no follow-up call was required.

### Data analysis

Quantitative data from closed questions were analysed using descriptive statistics with standard statistical software (Prism 10). Free-text answers were used to contextualise and illuminate quantitative responses.

## Results

### Patient characteristics

**Figure 1:**
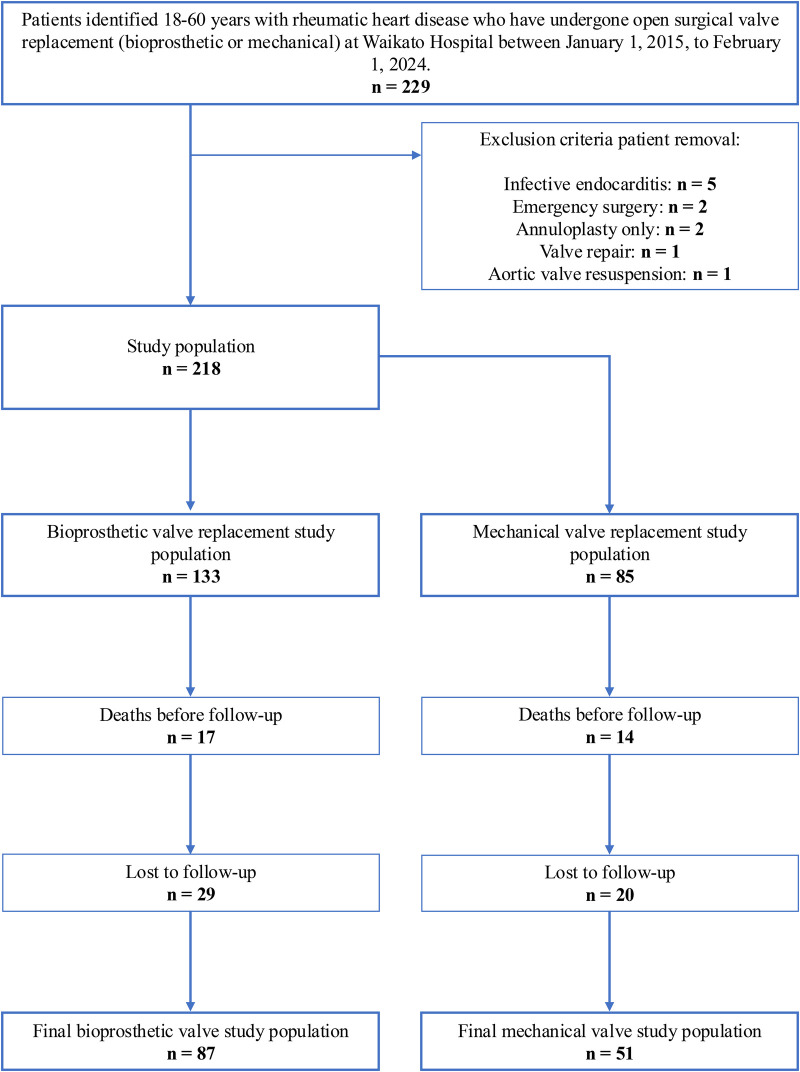
Study population flowchart of rheumatic heart disease patients who had undergone valve replacement surgery from January 1, 2015 to December 31, 2020.

229 adult patients with RHD underwent open surgical valve replacement or repair at Waikato Hospital between 1st January 2015 and 31st December 2020 (Figure 1). After applying the exclusion criteria, 218 patients formed the final study population. 31 (14%) died prior to follow-up (causes of death are listed in: Supplementary Appendix Table 1), and 49 (22%) patients were lost to follow-up as they were uncontactable despite multiple attempts. 187 patients were approached, and 138 patients completed or partially completed the survey (four patients partially completed the survey, three with a bioprosthetic prosthesis and one with a mechanical prosthesis; the denominator is subject to change; however, this is specified at the beginning of each section).

**Figure 2:**
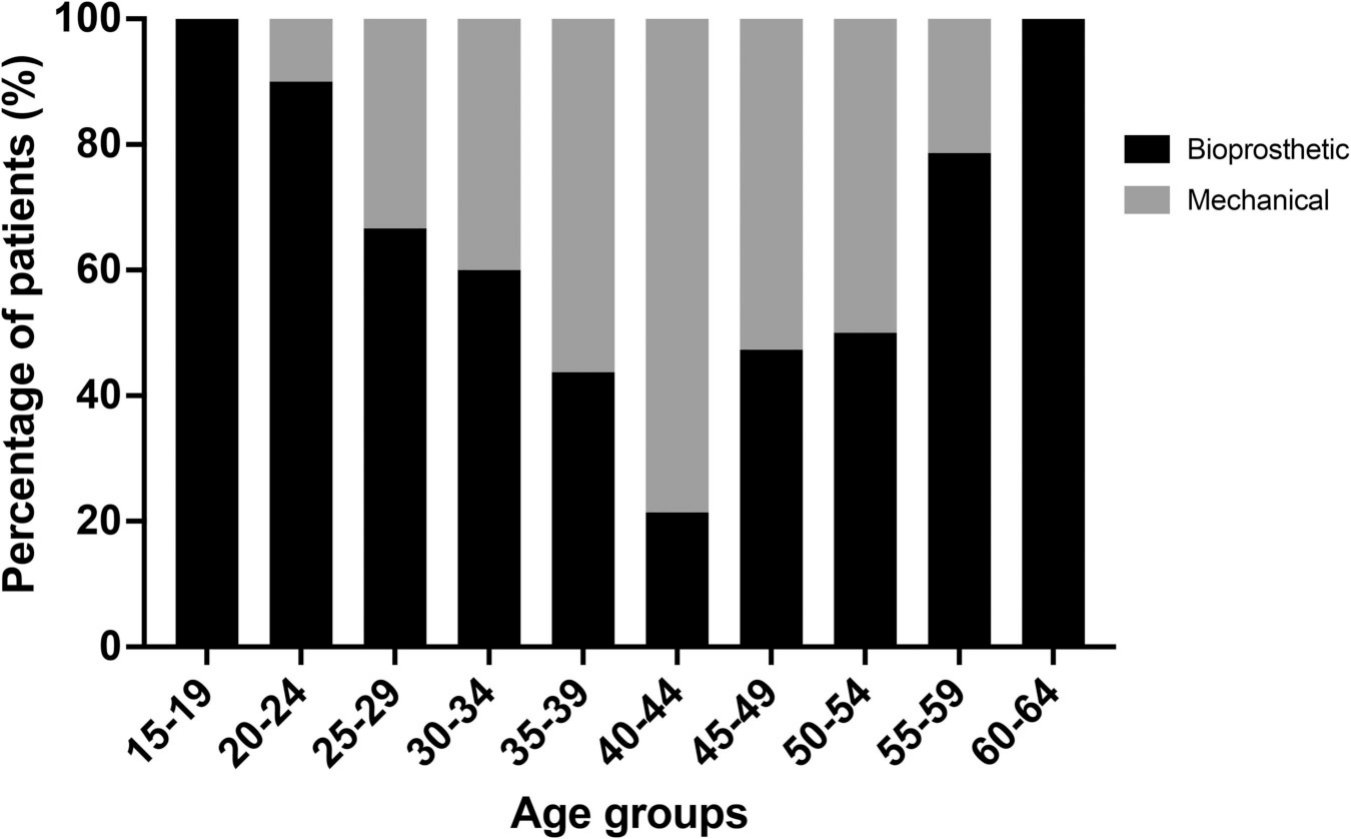
Bar graph of valve implant type (bioprostheric or mechanical) stratified by age group. Results are shown as a percentage of total patients for the age group; the total number of patients included is 218 (full study population).

Table 1 describes the baseline demographics of the cohort (total study population of 218 patients). The mean age at the time of the operation was 49 (12) years, and 98 (45%) patients were female. The age distribution was predominantly 45–49 years (38, 17%), followed by 50–54 years (54, 25%) and 55–59 years (61, 28%). The ethnicity of the majority of patients was either NZ European (103, 47%) or Māori (97, 44%) and 125 (57%) patients lived in NZ index of deprivation deciles 7–10, representing the most deprived socioeconomic areas. Operations were dominated by single valve replacements (166, 76%), predominantly aortic valve replacements (108, 50%). 31 (15%) patients died prior to follow-up. Bioprosthetic valves were implanted in 133 (61%) patients, and mechanical valves in 85 (39%) patients. The mean follow-up period (time from operation date to telephone survey) for patients who answered (138 patients) was 6.1 ± 1.8 years (minimum was 3.0 years, maximum was 8.9 years). Younger and older patients were more likely to choose bioprosthetic valves, whereas middle-aged patients between 35 and 50 years of age were more likely to have mechanical valves implanted from the total 218 patient population (Figure 2).

**Table 1:** Baseline characteristics.

Study population	Bioprosthetic	Mechanical	Total
	*n* = 133	*n* = 85	*n* = 218
Age at operation, mean ± SD	49 ± 12	47 ± 8	49 ± 10
Female, *n* (%)	54 (41%)	44 (52%)	98 (45%)
Age distribution			
15–19	2 (2%)	0 (0%)	2 (1%)
20–24	9 (7%)	1 (1%)	10 (6%)
25–29	4 (3%)	2 (2%)	6 (3%)
30–34	3 (2%)	2 (2%)	5 (2%)
35–39	7 (5%)	9 (11%)	16 (7%)
40–44	3 (2%)	11 (13%)	14 (6%)
45–49	18 (14%)	20 (24%)	38 (17%)
50–54	27 (20%)	27 (32%)	54 (25%)
55–59	48 (36%)	13 (15%)	61 (28%)
60–64	12 (9%)	0 (0%)	12 (6%)
Ethnicity			
NZ European	66 (50%)	37 (44%)	103 (47%)
Māori	56 (42%)	41 (48%)	97 (44%)
Pacific Islander	7 (5%)	6 (7%)	13 (6%)
Asian	3 (2%)	1 (1%)	4 (2%)
Other	0 (0%)	1 (1%)	1 (0.5%)
Operation type			
AVR, *n* (%)	78 (59%)	30 (35%)	108 (50%)
AVR/MVR, *n* (%)	17 (13%)	14 (16%)	31 (14%)
AVR/MVR/TVR, *n* (%)	2 (2%)	4 (5%)	6 (3%)
AVR/TVR, *n* (%)	1 (1%)	0 (0%)	1 (0.5%)
MVR, *n* (%)	26 (20%)	32 (38%)	58 (27%)
MVR/TVR, *n* (%)	9 (7%)	5 (6%)	14 (6%)
Decile			
1–2, *n* (%)	11 (8%)	5 (6%)	16 (10%)
3–4, *n* (%)	20 (15%)	10 (12%)	30 (14%)
5–6, *n* (%)	20 (15%)	11 (13%)	31 (14%)
7–8, *n* (%)	26 (20%)	22 (26%)	48 (22%)
9–10, *n* (%)	56 (42%)	37 (44%)	93 (43%)
Reoperation, *n* (%)	7 (8%)	1 (2%)	8 (6%)
Deaths	17 (12%)	14 (19%)	31 (14%)
Lost to follow-up	29 (22%)	20 (22%)	49 (22%)
	Bioprosthetic	Mechanical	Total
Final study population	*n* = 87	*n* = 51	*n* = 138
Mean follow-up period, mean ± SD (years)	6.0 ± 1.6	6.3 ± 2.0	6.1 ± 1.8

AVR, aortic valve replacement; MVR, mitral valve replacement; TVR, tricuspid valve replacement.

Four patients (three bioprosthetic valve and one mechanical valve patients) completed partial surveys of varying degrees; this may be reflected in changes to the final study population (*n*) across the various data sets.

### Preoperative factors that influenced prosthetic heart valve choice

Table 2 describes the primary reason for valve choice stratified by valve type (from 136 patients who completed the survey; two patients did not participate in this section). The most common reason for choosing a bioprosthetic valve was avoidance of warfarin (38, 44%), and for a mechanical valve was avoidance of re-operative cardiac surgery (31, 62%). Of importance, 15 (11%) patients stated that their valve choice was based on the surgeon's advice, and 9 (7%) stated they were not given any choice.

**Table 2:** Primary reason for choosing valve implant type (bioprosthetic or mechanical) as reported by patients.

	Bioprosthetic	Mechanical	Total
	*n* = 86	*n* = 50	*n* = 136
Information that influenced the final choice for valve implant type			
No warfarin, *n* (%)	38 (44%)	0 (0%)	38 (28%)
Risk of reoperation with bioprosthetic valve, *n* (%)	0 (0%)	31 (62%)	31 (23%)
Surgeon's advice, *n* (%)	11 (13%)	4 (8%)	15 (11%)
No choice given, *n* (%)	4 (5%)	5 (10%)	9 (7%)
Cardiologist's advice, *n* (%)	1 (1%)	4 (8%)	5 (4%)
Loud sounds of mechanical valve not wanted, *n* (%)	5 (6%)	0 (0%)	5 (4%)
Other, *n* (%)	27 (31%)	6 (12%)	33 (24%)

Patients’ answers lost due to no answer during the interview are excluded (one patient from each valve implant type, bioprosthetic and mechanical).

**Figure 3:**
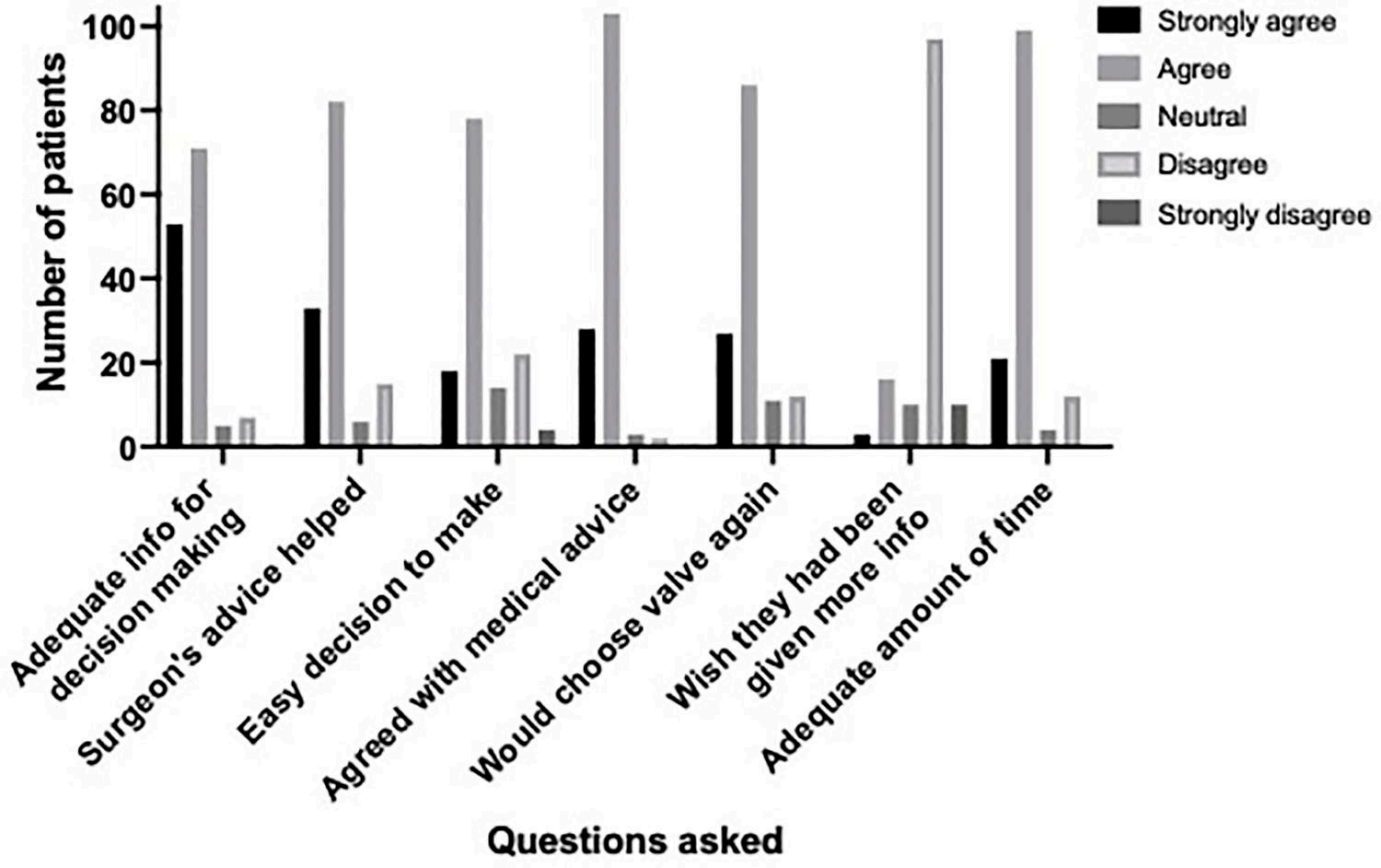
Bar graph of patients' opinions on the decision-making process of choosing their valve implant type. Results of Figure 3 are shown as a frequency of the answers given for the questions from the 136 patients who answered the questions. Patients' answers lost due to no answer during the interview are excluded (one patient from each valve implant type, bioprosthetic and mechanical).

Figure 3 illustrates patients' experience of the decision-making process prior to surgery.

Most patients (from 136 patients who completed the survey; two patients did not participate in this section) either “agreed” or 'strongly agreed' that they had been given adequate information for decision making (124, 91%), that the surgeon's advice was helpful (115, 85%), that it was an easy decision to make (96, 76%), that they would choose the same valve (113, 83%) and that they had been given an adequate amount of time to make the decision (130, 96%).

Surgeons (70, 48%) and cardiologists (51, 35%) were perceived as the most helpful information providers concerning valve choice, whereas primary care doctors (17, 33%) and cardiologists (14, 27%) were the most helpful for warfarin education (from 136 patients who completed the survey; two patients did not participate in this section; patients could give multiple answers) (Supplementary Appendix Table 2).

**Figure 4:**
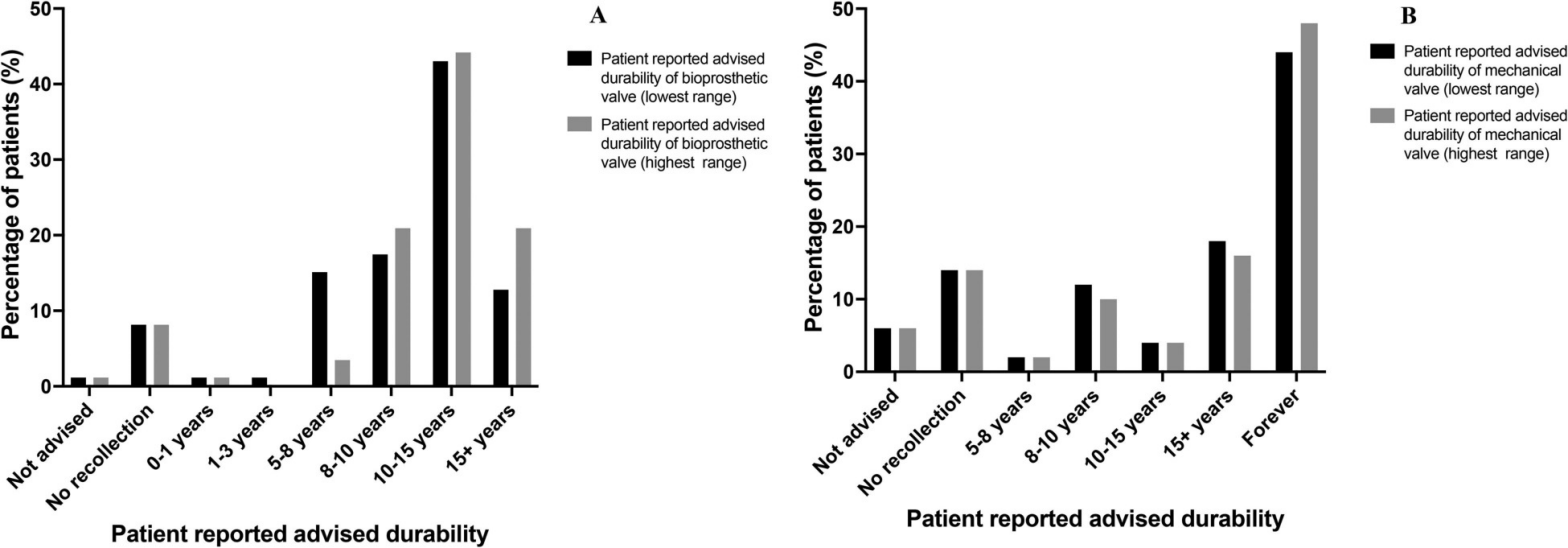
Bar graphs of the lowest and highest range of the advised durability of either bioprosthetic **(A)** or mechanical **(B)** valve as reported by patients. Results are shown as percentages of the 136 patients who answered the questions. Patients' answers lost due to no answer during the interview are excluded (one patient from each valve implant type, bioprosthetic and mechanical).

Figure 4 illustrates the patient-reported advised durability for each valve type (from 136 patients who completed the survey; two patients did not participate in this section). 37 (44%) of bioprosthetic valve patients were advised that their valve would last 10-15 or 15+ years. For mechanical valve patients, the most popular answer was that the valve would last “forever” (22, 44%).

### Postoperative experience with prosthetic heart valves and anticoagulation

Seventy-eight (90%) patients with a bioprosthetic valve and 41 (80%) patients with a mechanical valve stated they would choose the same valve type again. Table 3 describes the positive and negative patient perceptions of either valve type (from 136 patients who completed the survey; two patients did not participate in this section). 66 (77%) patients stated no negative experiences with having a bioprosthetic valve, whereas only 23 (46%) patients stated no negative experiences with having a mechanical valve. Taking warfarin and the clicking sound of the valve were the most common negatives described by patients receiving a mechanical valve.

**Table 3:** Positive and negative patient perceptions of bioprosthetic and mechanical valves.

	Bioprosthetic	Mechanical
	*n* = 86	*n* = 50
Negatives of having a bioprosthetic valve		
No negatives, *n* (%)	66 (77%)	
Deteriorating fast, *n* (%)	3 (3%)	
Has shortness of breath, *n* (%)	3 (3%)	
Other, *n* (%)	14 (16%)	
Positives of having a bioprosthetic valve		
No positives, *n* (%)	51 (59%)	
Improved fitness, *n* (%)	20 (23%)	
Other, *n* (%)	9 (10%)	
Can live active lifestyle, *n* (%)	6 (7%)	
Negatives of having a mechanical valve		
No negatives, *n* (%)		23 (46%)
Taking warfarin, *n* (%)		13 (26%)
Loud sounds, *n* (%)		12 (24%)
Other, *n* (%)		2 (4%)
Positives of having a mechanical valve		
No positives, *n* (%)		39 (78%)
Improved fitness, *n* (%)		8 (16%)
No shortness of breath, *n* (%)		1 (2%)
Other, *n* (%)		2 (4%)

Patients’ answers lost due to no answer during the interview are excluded (one patient from each valve implant type, bioprosthetic and mechanical).

Amongst mechanical valve patients (from 50 mechanical valve patients who completed the survey; one patient did not participate in this section), 28 (56%) agreed that warfarin had impacted their life, although 14 (28%) disagreed with this statement. However, the majority of patients stated that taking warfarin was better than expected (28, 56%), and 13 (26%) disagreed with this statement (Supplementary Appendix Figure 1). Table 4 describes the number of bleeding and clotting events from warfarin use and difficulties obtaining INR blood tests. Concerning bleeding events, 41 (82%) patients described no bleeding or thrombotic events and 9 (18%) patients described experiencing a significant bleeding/clotting event. The majority of patients stated no difficulties obtaining an INR blood test.

**Table 4:** Patients who have had bleeding or clotting events when taking warfarin and difficulties sorting warfarin levels as reported by patients.

	Mechanical
Population, *n*	50
Bleeding/clotting events due to warfarin use	
None, *n* (%)	41 (82%)
Bleeding, *n* (%)	7 (14%)
Clotting, *n* (%)	2 (4%)
Difficulties obtaining an INR blood test	
No difficulties, *n* (%)	48 (96%)
Stroke during surgery, lost eyesight, relies on someone else, *n* (%)	1 (2%)
Travel, *n* (%)	1 (2%)

Patients’ answers lost due to no answer during the interview are excluded (one patient from mechanical valve implant type).

### Patients' knowledge of anticoagulation

In terms of patient warfarin management strategies (from 51 mechanical valve patients taking warfarin), 34 (67%) reported having an INR range of 2.5–3.5, with over 50% of patients having an INR test once a month. A small proportion reported INR testing frequency at six weeks, two months and three months. GPs were the most frequent managers of warfarin (31 61%), with pharmacists managing 15 (29%) patients (Supplementary Appendix Figure 2).

**Figure 5:**
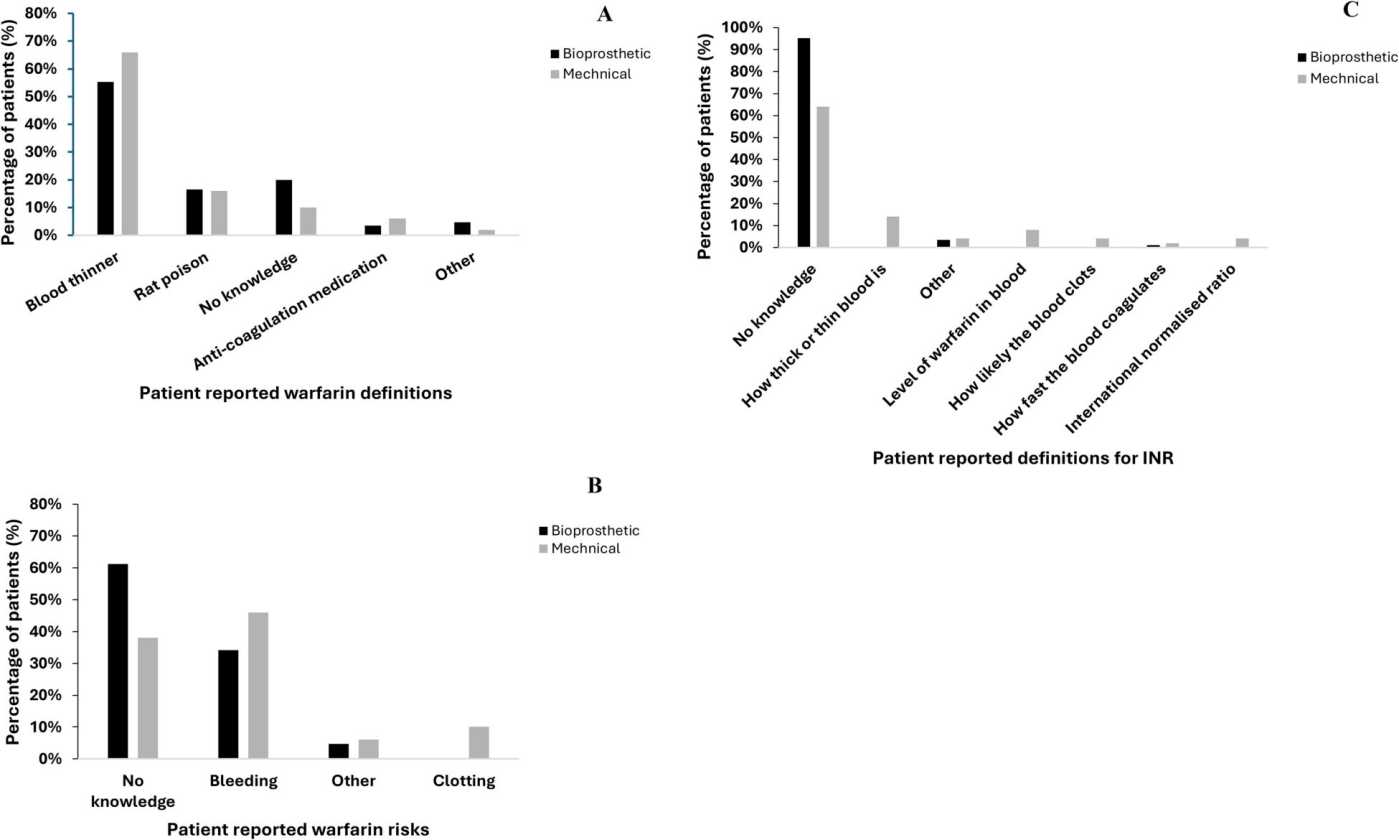
Bar graphs of patients' reported function/definition of warfarin **(A)**, their knowkdge of its risks **(B)**, and the definition of INR **(C)**. Results are shown as percentages of the 135 patients who answered the questions. Patients' answers lost due to no answer during the interview are excluded (two patients from the bioprosthetic valve category and one patient from the mechanical valve category).

Figure 5 illustrates patients' knowledge of anticoagulation in both bioprosthetic and mechanical valve patients (from 135 patients who completed the survey; three patients did not participate in this section). Regarding the definition of warfarin, 80 (59%) patients stated that warfarin was a blood thinner, and 22 (16%) patients stated they had no knowledge. Concerning risks associated with warfarin, 71 (53%) stated they had no knowledge of the risks, and 52 (39%) stated bleeding as a risk. Concerning the definition of INR, 113 (84%) stated they had no knowledge of the definition of INR.

Looking at patients' dental management (from 137 patients who completed the survey; one patient did not participate in this section), 63 (46%) reported their dental health as excellent or good, whereas 24 (18%) patients reported it as poor or very poor. 64 (47%) patients reported they had never seen a dentist since the operation, and 25 (18%) stated the main difficulty preventing them from seeing a dentist was cost (Supplementary Appendix Figure 3).

Post-operatively, (from 136 patients who completed the survey; two patients did not participate in this section), 46 (34%) patients had not seen a medical practitioner since their valve replacement; however, 95 (70%) reported having no difficulties in seeing a medical practitioner. The most common medical practitioner seen was a cardiologist post-operatively by 66 (49%) of patients (Supplementary Appendix Figure 4).

The majority of patients (132, 97%) did not receive prophylactic penicillin injections following surgery (from 136 patients who completed the survey; two patients did not participate in this section) (Supplementary Appendix Figure 5).

**Figure 6:**
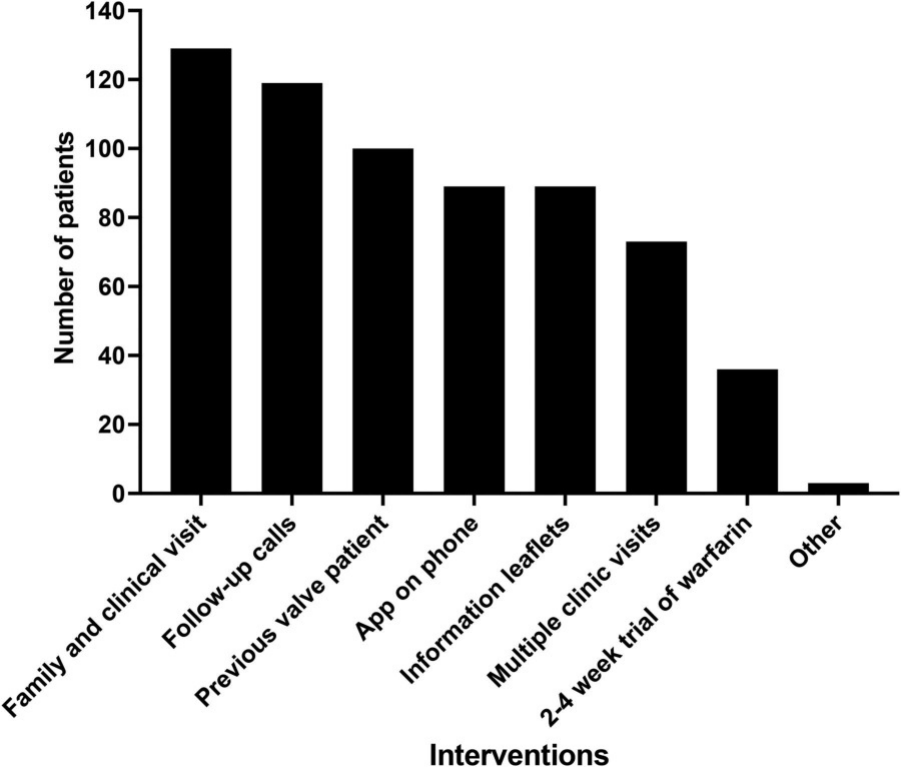
Bar graph of patient-reported suggestions for interventions and education regarding value implant type and warfarin. Rsults are shown as frequencies of the 136 patients who answered the questions. Patients whose answer were lost due to no answer during the interview are excluded (two patients from the bioprosthetic valve category).

Concerning suggestions to improve valve choice and education (from 136 patients who completed the survey; two patients did not participate in this section), over 50% of patients suggested involving family at clinical visits, follow-up calls, having the opportunity to speak to previous valve patients, having an app on the phone with information, provision of information leaflets, having the opportunity for additional preoperative clinic visits and a preoperative warfarin trial would help (Figure 6).

## Discussion

### Main findings

This is one of the first studies to examine patient choice and experience with valve prosthesis and anticoagulation in an adult population with RHD. Our main findings are: 1) Over 60% of RHD patients less than 60 years of age undergoing valve replacement received a bioprosthetic valve, 2) Surgeons and cardiologists were the most influential information providers in a patient's valve prosthesis decision making process, and general practitioners are perceived as most important in a patient's anticoagulation management, 3) Over 40% of RHD patients with bioprosthetic valves stated their valve durability is greater than 10–15 years, 4) Although warfarin did impact the lives of patients with mechanical valves, the majority stated that the experience of anticoagulation was better than expected, 5) Long term dental management is poor with 47% of patients never seeing a dentist after surgery, 6) Almost all patients discontinued prophylactic penicillin following discharge.

Our study demonstrates that patient education and valve choice remain unsatisfactory, with poor knowledge of anticoagulation, lack of clarity of valve durability, particularly with bioprosthetic valves in young patients and insufficient information to make informed valve choices. This reflects existing data in patients with degenerative valve disease, where up to 70% of patients on warfarin have been found to have insufficient knowledge of their therapy; in addition, warfarin knowledge has been positively associated with better INR control (7). Similarly, in 132 patients with degenerative aortic valve disease, 56% experienced decisional conflict, 68% wanted to be involved in decision-making, only 53% agreed that they were actually involved, and only 69% were able to answer three basic questions concerning their prosthetic valve (8). Our study extends these findings in a younger population with RHD.

Shared decision-making is a collaborative process between clinicians who provide information on prosthesis options, whilst incorporating patient values and preferences into the final decision. Our findings that surgeons and cardiologists were the most influential when providing information imply that specialists are the primary point of communication when patients require access to clinical information to make a meaningful decision. True decision-making, however, requires that patients be able to comprehend the information, deliberate, and feel involved. The knowledge gaps identified, including misconceptions around bioprosthetic valve durability and anticoagulation literacy, suggest that information exchange alone is insufficient for comprehensive shared decision-making. The quality of the decision in the context of valve prosthesis is health literacy, awareness that more than one option is available, having support people, and adequate communication in different forms (9).

Our patients provided suggestions for improving communication, such as inviting family members to clinic visits, providing additional clinic visits, app development, etc. Interventions, such as online patient decision aids, help improve patient knowledge and mental well-being regarding valve selection as demonstrated in a randomised controlled trial (10).

Dental care is essential post-valve replacement to minimise the risk of infective endocarditis complications. However, our data shows that a significant portion of patients are not receiving long-term dental care to minimise the risk of reintervention being required. This can be attributed to the unaffordability of dental care, as demonstrated in a New Zealand survey, where 44% of adult participants reported costs as the largest factor that has prevented access to dental care, with Māori and Pasifika most affected. The cost of dental care has risen disproportionately compared to other services in the last few decades (11).

Nearly all patients also stopped receiving secondary prophylactic penicillin injections post-discharge. Although there may be a small risk of having recurrent rheumatic fever post-valve replacement, a study looking specifically at RHD patients who had undergone a valve replacement from 1990 to 2014 found no statistically significant difference in clinical outcomes of patients who had secondary prophylaxis and those who didn't (12).

### Strengths and limitations

This study has several strengths; it is the first study to describe the experiences of RHD patients with valve choice and anticoagulation. The survival follow-up data is robust and complete as it is obtained by linkage to national minimum datasets concerning mortality at Health New Zealand. This study has some limitations. The study was retrospective, self-reported and based on patient recall. This introduces recall bias; patients may not have accurately remembered information.

Additionally, patient-reported perspectives may have been influenced by cognitive dissonance, whereby individuals rationalise their treatment decisions favourably despite associated drawbacks. Rheumatic heart disease (RHD) is disproportionately prevalent among populations of lower socioeconomic status, which may contribute to reduced patient contactability and an increased risk of complications due to limited access to healthcare services (13). These factors introduce the potential for both attrition bias and ascertainment bias within the study.

Although rheumatic heart disease is classically described as predominantly affecting the mitral valve, the higher proportion of aortic valve replacements (AVRs) compared with mitral valve replacements (MVRs) observed in this cohort does not necessarily imply inclusion of degenerative aortic valve disease. The observed distribution is likely to reflect differences in disease progression and management strategies rather than true underlying prevalence. The NZ RHD registry (14) demonstrates that the burden of mixed mitral and aortic valve disease and of aortic valve disease alone increases in adulthood, consistent with the age profile of our cohort. In addition, percutaneous balloon mitral intervention is frequently utilised in New Zealand (most frequently in isolated mitral valve disease), and rheumatic mitral valve disease is often managed with repair, as evidenced by better long-term outcomes with equivalent durability to MVR (15). These factors limit the progression to requiring an MVR. In contrast, rheumatic aortic valve disease, particularly when presenting with significant regurgitation, is less frequently amenable to repair and more commonly requires valve replacement (16). Consequently, referral and intervention bias are likely present, such that this cohort reflects patients undergoing valve replacement rather than the full spectrum of rheumatic valve disease within the population.

## Conclusion

This study highlights the importance of optimising shared decision-making. Most patients were satisfied with the process; however, the need for comprehensive pre-operative education and post-operative ongoing support is evident. Efforts in tailoring education resources to patients are required to obtain complete, informed decision-making and improve long-term patient outcomes from valve replacement surgery in young patients with RHD.

## Data Availability

The original contributions presented in the study are included in the article, further inquiries can be directed to the corresponding author/s.
