# Supplementary material for: Patient perspectives of prosthetic heart valve choice and anticoagulation in patients with rheumatic heart disease: a semi-quantitative study
Source: Front Cardiovasc Med. 2026 Jun 8;13:1810992. doi: 10.3389/fcvm.2026.1810992 (PMC13283989; doi:10.3389/fcvm.2026.1810992)
Supplement: Supplementary file 1 [file Table1.docx]

***Appendix Table 1: Causes of death of patients unable to take part in follow-up.***

|  | **Bioprosthetic** | **Mechanical** | **Total** |
| --- | --- | --- | --- |
|  | n = 17 | n = 14 | n = 31 |
| **Causes of death** |  |  |  |
| Unknown, n (%) | 8 (47%) | 6 (43%) | 14 (45%) |
| Cardiac failure, n (%) | 0 (0%) | 3 (21%) | 3 (10%) |
| Cardiac failure directly related to prosthesis, n (%) | 2 (12%) | 1 (7%) | 3 (10%) |
| Infective endocarditis, n (%) | 1 (6%) | 0 (0%) | 1 (3%) |
| Sepsis secondary to pneumonia, n (%) | 1 (6%) | 0 (0%) | 1 (3%) |
| **Post-operative death** |  |  |  |
| Cardiac failure, n (%) | 3 (18%) | 2 (14%) | 5 (16%) |
| Embolic ischaemic stroke, n (%) | 1 (6%) | 0 (0%) | 1 (3%) |
| Acute respiratory distress, n (%) | 0 (0%) | 1 (7%) | 1 (3%) |
| Haematoma of the descending aorta, n (%) | 1 (6%) | 0 (0%) | 1 (3%) |
| Acute myocardial infarction, n (%) | 0 (0%) | 1 (7%) | 1 (3%) |

Unknown causes present due to a lack of documentation.

***Appendix Table 2: Most helpful information providers reported by patients for valve implant types and warfarin medication.***

|  | **The most helpful information providers concerning valve implant types** | **The most helpful information providers concerning warfarin** |
| --- | --- | --- |
| Surgeon, n (%) | 70 (48%) | 5 (10%) |
| Cardiologist, n (%) | 51 (35%) | 14 (27%) |
| No recollection, n (%) | 14 (10%) | 3 (6%) |
| General practitioner, n (%) | 3 (2%) | 17 (33%) |
| Anaesthesiologist, n (%) | 2 (1%) | 0 (0%) |
| Family, n (%) | 2 (1%) | 2 (4%) |
| No information given, n (%) | 2 (1%) | 1 (2%) |
| Pharmacist, n (%) | 1 (1%) | 5 (10%) |
| Previous valve patients, n (%) | 1 (1%) | 0 (0%) |
| Personal research, n (%) | 0 (0%) | 2 (4%) |
| Nurse, n (%) | 0 (0%) | 2 (4%) |
| Friend, n (%) | 0 (0%) | 0 (0%) |
| **Total frequency, f** | **146** | **51** |

Patients’ answers lost due to no answer during the interview are excluded (One patient from each valve implant type, bioprosthetic and mechanical). 136 patients answered.

***Appendix Figure 1: Bar graph of patients' opinions on their experience with warfarin.*** Results of Appendix Figure 1 are shown as a frequency of the answers given for the questions from 50 patients who answered the questions (One mechanical valve patient did not participate in this section).

**C**

**A**

**B**

**D**

***Appendix Figure 2: Bar graphs of patient-reported data of warfarin INR ranges (2A), INR blood test frequency (2B), warfarin dosage management (2C) and INR blood test location (2D).*** Results are shown as percentages of the 51 patients who answered the questions and have a mechanical valve.

**C**

**A**

**B**

***Appendix Figure 3: Bar graphs of patients’ reported dental health rating (3A), frequency of dental health visits (3B) and difficulties seeing a dental practitioner (3C).*** Results are shown as percentages and frequencies of the 137 patients who answered the questions. Patients whose answers were lost due to no answer during the interview are excluded (One patient from the bioprosthetic valve category).

**C**

**A**

**B**

***Appendix Figure 4: Bar graphs of patients’ reported medical practitioner frequency of visits (4A), practitioner visited (4B) and difficulties seeing a medical practitioner concerning their heart (4C).*** Results are shown as percentages and frequencies of the 136 patients who answered the questions. Patients whose answers were lost due to no answer during the interview are excluded (Two patients from the bioprosthetic valve category).

******

***Appendix Figure 5: Patients receiving penicillin injections as reported by patients.*** Results are shown as a percentage of the 136 patients who answered the questions. Patients whose answers were lost due to no answer during the interview are excluded (Two patients from the bioprosthetic valve category).
